# Supplementary material for: Hot spot prediction in protein-protein interactions by an ensemble system
Source: BMC Syst Biol. 2018 Dec 31;12(Suppl 9):132. doi: 10.1186/s12918-018-0665-8 (PMC6311905; doi:10.1186/s12918-018-0665-8)
Supplement: Supplementary file 2 — All relevant MATLAB code. A simple Matlab implement of our predictor. (ZIP 19,742 kb) [file 12918_2018_665_MOESM2_ESM.zip › Additional file 2/Read ASA/ASA website/dbMPIKT/3NGBC.html]

 NetSurfP - Protein Surface Accessibility and Secondary Structure Predictions

  

|  |  |  |
| --- | --- | --- |
|  |  | NetSurfP - Protein Surface Accessibility and Secondary Structure Predictions  Technical University of Denmark |

  


---

```
# For publication of results, please cite:
# A generic method for assignment of reliability scores applied to solvent accessibility predictions.
# Bent Petersen, Thomas Nordahl Petersen, Pernille Andersen, Morten Nielsen and Claus Lundegaard
# BMC Structural Biology 2009, 9:51 doi:10.1186/1472-6807-9-51
#
# Column 1: Class assignment - B for buried or E for Exposed - Threshold: 25% exposure, but not based on RSA
# Column 2: Amino acid
# Column 3: Sequence name
# Column 4: Amino acid number
# Column 5: Relative Surface Accessibility - RSA
# Column 6: Absolute Surface Accessibility
# Column 7: Z-fit score for RSA prediction
# Column 8: Probability for Alpha-Helix
# Column 9: Probability for Beta-strand
# Column 10: Probability for Coil
E E  3NGBC                  1    0.793 138.624   0.766   0.003   0.003   0.994
B I  3NGBC                  2    0.235  43.549  -0.475   0.003   0.718   0.279
E V  3NGBC                  3    0.414  63.693   1.339   0.001   0.900   0.099
B L  3NGBC                  4    0.124  22.704   0.474   0.001   0.900   0.099
E T  3NGBC                  5    0.407  56.437   0.351   0.002   0.816   0.182
B Q  3NGBC                  6    0.149  26.683   0.562   0.004   0.514   0.481
E S  3NGBC                  7    0.363  42.544   0.106   0.004   0.138   0.858
E P  3NGBC                  8    0.472  66.920  -1.184   0.018   0.019   0.964
E G  3NGBC                  9    0.524  41.262  -1.237   0.018   0.088   0.893
E T  3NGBC                 10    0.359  49.793  -1.391   0.021   0.451   0.528
B L  3NGBC                 11    0.258  47.258  -0.214   0.021   0.756   0.223
E S  3NGBC                 12    0.474  55.529  -0.841   0.018   0.846   0.136
B L  3NGBC                 13    0.247  45.152   0.337   0.021   0.756   0.223
E S  3NGBC                 14    0.419  49.072   0.424   0.004   0.420   0.576
E P  3NGBC                 15    0.514  72.894  -0.111   0.004   0.085   0.910
E G  3NGBC                 16    0.676  53.162  -0.959   0.005   0.015   0.979
E E  3NGBC                 17    0.508  88.800   0.391   0.004   0.138   0.858
E T  3NGBC                 18    0.525  72.817   0.948   0.004   0.616   0.381
B A  3NGBC                 19    0.064   7.086  -0.119   0.001   0.900   0.099
E I  3NGBC                 20    0.443  81.900   1.584   0.001   0.959   0.040
B I  3NGBC                 21    0.050   9.268   0.539   0.001   0.959   0.040
E S  3NGBC                 22    0.358  41.922   1.040   0.001   0.900   0.099
B C  3NGBC                 23    0.031   4.296  -0.046   0.001   0.900   0.099
E R  3NGBC                 24    0.421  96.317   1.105   0.001   0.900   0.099
B T  3NGBC                 25    0.183  25.382  -1.252   0.003   0.718   0.279
E S  3NGBC                 26    0.364  42.708  -0.895   0.005   0.336   0.660
E Q  3NGBC                 27    0.610 108.910  -2.391   0.005   0.045   0.951
E Y  3NGBC                 28    0.448  95.802  -1.953   0.005   0.045   0.951
B G  3NGBC                 29    0.287  22.595  -0.929   0.019   0.141   0.840
B S  3NGBC                 30    0.211  24.741  -0.251   0.022   0.552   0.426
B L  3NGBC                 31    0.041   7.580  -0.667   0.021   0.756   0.223
B A  3NGBC                 32    0.114  12.596   0.137   0.018   0.846   0.136
B W  3NGBC                 33    0.041   9.909   0.334   0.018   0.846   0.136
B Y  3NGBC                 34    0.074  15.899   0.178   0.011   0.918   0.071
B Q  3NGBC                 35    0.169  30.183  -0.041   0.018   0.846   0.136
B Q  3NGBC                 36    0.166  29.737   0.324   0.004   0.514   0.481
E R  3NGBC                 37    0.445 101.859   1.506   0.004   0.085   0.910
E P  3NGBC                 38    0.599  85.055   0.016   0.005   0.015   0.979
E G  3NGBC                 39    0.817  64.266  -2.245   0.003   0.003   0.994
E Q  3NGBC                 40    0.464  82.924   0.599   0.018   0.047   0.935
E A  3NGBC                 41    0.369  40.653  -0.101   0.018   0.088   0.893
B P  3NGBC                 42    0.110  15.552   0.060   0.005   0.336   0.660
E R  3NGBC                 43    0.307  70.280   0.589   0.002   0.816   0.182
B L  3NGBC                 44    0.153  27.996   0.288   0.001   0.900   0.099
B V  3NGBC                 45    0.054   8.331   0.670   0.001   0.959   0.040
B I  3NGBC                 46    0.042   7.770  -0.627   0.001   0.959   0.040
B Y  3NGBC                 47    0.123  26.200  -0.200   0.001   0.959   0.040
B S  3NGBC                 48    0.157  18.459  -1.091   0.001   0.900   0.099
B G  3NGBC                 49    0.169  13.324  -1.827   0.004   0.514   0.481
B S  3NGBC                 50    0.273  32.031  -1.310   0.020   0.205   0.775
E T  3NGBC                 51    0.561  77.811  -1.902   0.019   0.141   0.840
E R  3NGBC                 52    0.412  94.394  -0.754   0.018   0.047   0.935
E A  3NGBC                 53    0.572  63.012  -2.676   0.018   0.047   0.935
E A  3NGBC                 54    0.670  73.867  -1.613   0.018   0.047   0.935
E G  3NGBC                 55    0.580  45.638  -1.462   0.018   0.088   0.893
B I  3NGBC                 56    0.256  47.415  -0.054   0.018   0.088   0.893
E P  3NGBC                 57    0.543  77.038  -0.797   0.004   0.085   0.910
E D  3NGBC                 58    0.539  77.612  -2.240   0.004   0.138   0.858
E R  3NGBC                 59    0.348  79.806  -0.394   0.005   0.336   0.660
B F  3NGBC                 60    0.120  24.004  -1.511   0.004   0.616   0.381
B S  3NGBC                 61    0.266  31.222  -1.069   0.023   0.655   0.322
B G  3NGBC                 62    0.264  20.745  -2.543   0.022   0.552   0.426
E S  3NGBC                 63    0.379  44.454  -1.244   0.004   0.420   0.576
E R  3NGBC                 64    0.431  98.791  -1.305   0.005   0.336   0.660
E W  3NGBC                 65    0.439 105.652  -2.146   0.004   0.197   0.799
E G  3NGBC                 66    0.621  48.912  -2.260   0.004   0.085   0.910
E P  3NGBC                 67    0.452  64.110  -1.655   0.004   0.138   0.858
E D  3NGBC                 68    0.413  59.513  -0.495   0.004   0.616   0.381
B Y  3NGBC                 69    0.114  24.426  -0.474   0.003   0.718   0.279
B N  3NGBC                 70    0.224  32.779  -0.920   0.003   0.718   0.279
B L  3NGBC                 71    0.063  11.499  -0.205   0.003   0.718   0.279
B T  3NGBC                 72    0.187  25.951  -0.561   0.002   0.816   0.182
B I  3NGBC                 73    0.034   6.383  -1.136   0.021   0.756   0.223
B S  3NGBC                 74    0.246  28.878  -0.419   0.023   0.655   0.322
B N  3NGBC                 75    0.270  39.499  -1.533   0.021   0.451   0.528
B L  3NGBC                 76    0.144  26.330  -0.419   0.019   0.141   0.840
E E  3NGBC                 77    0.447  78.038  -0.016   0.018   0.088   0.893
E S  3NGBC                 78    0.471  55.201  -1.166   0.019   0.141   0.840
E G  3NGBC                 79    0.666  52.422  -1.931   0.018   0.088   0.893
E D  3NGBC                 80    0.545  78.592  -1.260   0.004   0.085   0.910
E F  3NGBC                 81    0.490  98.423  -0.892   0.005   0.262   0.733
B G  3NGBC                 82    0.193  15.165  -1.216   0.004   0.514   0.481
E V  3NGBC                 83    0.344  52.827   0.217   0.002   0.816   0.182
B Y  3NGBC                 84    0.116  24.768   0.707   0.001   0.900   0.099
B Y  3NGBC                 85    0.174  37.120   0.062   0.001   0.900   0.099
B C  3NGBC                 86    0.196  27.589  -0.258   0.002   0.816   0.182
B Q  3NGBC                 87    0.268  47.901  -0.301   0.023   0.655   0.322
B Q  3NGBC                 88    0.283  50.615  -0.645   0.021   0.279   0.699
B Y  3NGBC                 89    0.293  62.593  -1.601   0.021   0.279   0.699
E E  3NGBC                 90    0.361  63.119  -1.350   0.022   0.359   0.619
E F  3NGBC                 91    0.405  81.364  -1.671   0.020   0.205   0.775
E F  3NGBC                 92    0.388  77.872  -1.008   0.056   0.142   0.802
E G  3NGBC                 93    0.429  33.802  -1.548   0.018   0.047   0.935
E Q  3NGBC                 94    0.538  95.998  -1.377   0.018   0.047   0.935
E G  3NGBC                 95    0.421  33.172  -1.217   0.018   0.088   0.893
E T  3NGBC                 96    0.357  49.488  -1.069   0.018   0.088   0.893
E K  3NGBC                 97    0.326  67.058  -0.635   0.021   0.279   0.699
E V  3NGBC                 98    0.310  47.632  -0.823   0.022   0.359   0.619
E Q  3NGBC                 99    0.320  57.206  -1.269   0.021   0.451   0.528
E V  3NGBC                100    0.294  45.126  -1.106   0.022   0.552   0.426
E D  3NGBC                101    0.308  44.311  -0.584   0.021   0.451   0.528
E I  3NGBC                102    0.360  66.619  -1.237   0.021   0.279   0.699
E K  3NGBC                103    0.369  75.924  -1.455   0.056   0.142   0.802
E R  3NGBC                104    0.314  71.883  -1.164   0.019   0.141   0.840
E T  3NGBC                105    0.486  67.478  -1.538   0.019   0.141   0.840
E V  3NGBC                106    0.487  74.821  -1.707   0.019   0.141   0.840
E A  3NGBC                107    0.337  37.126  -1.625   0.018   0.047   0.935
E A  3NGBC                108    0.468  51.530  -2.036   0.004   0.085   0.910
E P  3NGBC                109    0.486  69.006  -1.673   0.004   0.197   0.799
E S  3NGBC                110    0.399  46.716  -1.345   0.022   0.359   0.619
B V  3NGBC                111    0.284  43.712  -1.050   0.021   0.451   0.528
B F  3NGBC                112    0.278  55.734  -1.652   0.021   0.451   0.528
E I  3NGBC                113    0.347  64.176  -1.779   0.005   0.336   0.660
E F  3NGBC                114    0.335  67.275  -1.600   0.004   0.138   0.858
E P  3NGBC                115    0.342  48.473  -1.894   0.005   0.045   0.951
B P  3NGBC                116    0.286  40.612  -2.399   0.018   0.019   0.964
B S  3NGBC                117    0.285  33.437  -2.449   0.018   0.019   0.964
E D  3NGBC                118    0.456  65.695  -2.076   0.018   0.047   0.935
E E  3NGBC                119    0.508  88.678  -1.988   0.053   0.043   0.903
E Q  3NGBC                120    0.326  58.224  -3.028   0.018   0.088   0.893
E L  3NGBC                121    0.357  65.312  -1.993   0.018   0.047   0.935
E K  3NGBC                122    0.537 110.399  -1.604   0.018   0.047   0.935
E S  3NGBC                123    0.655  76.743  -1.533   0.018   0.047   0.935
E G  3NGBC                124    0.592  46.583  -1.791   0.005   0.045   0.951
E T  3NGBC                125    0.434  60.237   0.340   0.005   0.336   0.660
B A  3NGBC                126    0.068   7.505  -0.567   0.002   0.816   0.182
B S  3NGBC                127    0.133  15.541   0.617   0.001   0.900   0.099
B V  3NGBC                128    0.031   4.703   0.378   0.001   0.959   0.040
B V  3NGBC                129    0.025   3.904   0.028   0.001   0.959   0.040
B C  3NGBC                130    0.015   2.036   1.380   0.001   0.959   0.040
B L  3NGBC                131    0.079  14.465   0.146   0.001   0.959   0.040
B L  3NGBC                132    0.024   4.468   0.479   0.002   0.816   0.182
B N  3NGBC                133    0.182  26.674   0.137   0.003   0.718   0.279
B N  3NGBC                134    0.271  39.660  -0.861   0.004   0.514   0.481
B F  3NGBC                135    0.274  55.092  -1.981   0.005   0.262   0.733
E Y  3NGBC                136    0.282  60.221  -0.260   0.004   0.197   0.799
E P  3NGBC                137    0.350  49.708  -1.676   0.004   0.197   0.799
E R  3NGBC                138    0.602 137.789  -1.924   0.004   0.138   0.858
E E  3NGBC                139    0.393  68.570  -0.684   0.004   0.420   0.576
B A  3NGBC                140    0.079   8.706   0.224   0.002   0.816   0.182
B K  3NGBC                141    0.223  45.830   0.364   0.001   0.900   0.099
B V  3NGBC                142    0.041   6.240   0.048   0.001   0.959   0.040
B Q  3NGBC                143    0.145  25.968   0.714   0.011   0.918   0.071
B W  3NGBC                144    0.039   9.355  -0.043   0.011   0.918   0.071
E K  3NGBC                145    0.248  50.952   0.815   0.001   0.900   0.099
E V  3NGBC                146    0.346  53.119   0.405   0.004   0.616   0.381
E D  3NGBC                147    0.674  97.167   0.217   0.005   0.045   0.951
E N  3NGBC                148    0.650  95.116  -1.299   0.005   0.015   0.979
E A  3NGBC                149    0.706  77.768  -0.796   0.004   0.138   0.858
E L  3NGBC                150    0.572 104.752  -1.440   0.005   0.262   0.733
E Q  3NGBC                151    0.376  67.082  -0.370   0.004   0.197   0.799
E S  3NGBC                152    0.493  57.815  -1.180   0.004   0.197   0.799
E G  3NGBC                153    0.515  40.523  -1.922   0.004   0.197   0.799
E N  3NGBC                154    0.461  67.432  -1.634   0.021   0.279   0.699
E S  3NGBC                155    0.307  35.969  -1.441   0.004   0.616   0.381
E Q  3NGBC                156    0.328  58.617  -1.041   0.021   0.756   0.223
E E  3NGBC                157    0.481  84.013  -1.166   0.023   0.655   0.322
E S  3NGBC                158    0.298  34.890  -1.610   0.022   0.359   0.619
E V  3NGBC                159    0.344  52.888  -0.684   0.022   0.359   0.619
E T  3NGBC                160    0.392  54.412  -0.922   0.022   0.359   0.619
E E  3NGBC                161    0.368  64.359  -0.684   0.021   0.279   0.699
E Q  3NGBC                162    0.475  84.889  -0.682   0.022   0.359   0.619
E D  3NGBC                163    0.579  83.463  -2.045   0.021   0.279   0.699
E S  3NGBC                164    0.601  70.484  -1.489   0.019   0.141   0.840
E K  3NGBC                165    0.524 107.849  -1.070   0.005   0.045   0.951
E D  3NGBC                166    0.728 104.962  -1.333   0.005   0.015   0.979
E S  3NGBC                167    0.466  54.662  -1.647   0.005   0.045   0.951
E T  3NGBC                168    0.308  42.720   0.218   0.004   0.420   0.576
B Y  3NGBC                169    0.087  18.571  -0.587   0.002   0.816   0.182
B S  3NGBC                170    0.095  11.181  -0.757   0.001   0.900   0.099
B L  3NGBC                171    0.063  11.590  -1.360   0.001   0.900   0.099
B S  3NGBC                172    0.119  13.947  -0.388   0.001   0.959   0.040
B S  3NGBC                173    0.042   4.934  -1.485   0.001   0.959   0.040
B T  3NGBC                174    0.183  25.354   0.127   0.001   0.959   0.040
B L  3NGBC                175    0.040   7.251  -0.133   0.001   0.900   0.099
E T  3NGBC                176    0.308  42.761   0.755   0.001   0.900   0.099
B L  3NGBC                177    0.057  10.437   0.470   0.003   0.718   0.279
E S  3NGBC                178    0.368  43.083   1.281   0.005   0.336   0.660
E K  3NGBC                179    0.481  98.859  -0.723   0.020   0.205   0.775
E A  3NGBC                180    0.566  62.329  -0.883   0.020   0.205   0.775
E D  3NGBC                181    0.529  76.229   0.878   0.020   0.205   0.775
B Y  3NGBC                182    0.176  37.526  -0.932   0.018   0.088   0.893
E E  3NGBC                183    0.654 114.236   0.325   0.005   0.045   0.951
E K  3NGBC                184    0.656 134.857   0.585   0.005   0.045   0.951
E H  3NGBC                185    0.631 114.688  -0.303   0.005   0.045   0.951
E K  3NGBC                186    0.504 103.735   0.364   0.005   0.262   0.733
E V  3NGBC                187    0.417  64.139   1.754   0.002   0.816   0.182
B Y  3NGBC                188    0.061  13.100   0.926   0.001   0.959   0.040
E A  3NGBC                189    0.304  33.556   1.726   0.001   0.959   0.040
B C  3NGBC                190    0.024   3.356   0.799   0.001   0.959   0.040
B E  3NGBC                191    0.265  46.261   1.358   0.001   0.959   0.040
B V  3NGBC                192    0.077  11.881  -0.264   0.001   0.959   0.040
E T  3NGBC                193    0.419  58.157   0.463   0.001   0.959   0.040
B H  3NGBC                194    0.147  26.685   0.372   0.002   0.816   0.182
E Q  3NGBC                195    0.651 116.197   0.035   0.005   0.262   0.733
E G  3NGBC                196    0.640  50.344  -1.869   0.004   0.085   0.910
E L  3NGBC                197    0.447  81.919  -0.942   0.004   0.085   0.910
E R  3NGBC                198    0.664 152.033  -0.089   0.004   0.085   0.910
E S  3NGBC                199    0.472  55.295   0.038   0.004   0.138   0.858
E P  3NGBC                200    0.448  63.600   0.238   0.004   0.420   0.576
B V  3NGBC                201    0.162  24.823  -0.628   0.002   0.816   0.182
E T  3NGBC                202    0.574  79.544   0.466   0.001   0.900   0.099
E K  3NGBC                203    0.272  55.909   1.300   0.001   0.900   0.099
E S  3NGBC                204    0.488  57.158   0.282   0.001   0.900   0.099
B F  3NGBC                205    0.101  20.311   0.890   0.001   0.900   0.099
E N  3NGBC                206    0.484  70.872   1.669   0.004   0.616   0.381
E R  3NGBC                207    0.468 107.058   0.530   0.020   0.205   0.775
E G  3NGBC                208    0.611  48.101  -1.442   0.019   0.141   0.840
E E  3NGBC                209    0.650 113.485   1.573   0.019   0.141   0.840
E C  3NGBC                210    0.509  71.534  -1.360   0.003   0.003   0.994

Explain the output.  Go back.
```

---
